# A comparative analysis of health status of international migrants and local population in Chile: a population-based, cross-sectional analysis from a social determinants of health perspective

**DOI:** 10.1186/s12889-022-13709-5

**Published:** 2022-07-12

**Authors:** Isabel Rada, Marcela Oyarte, Báltica Cabieses

**Affiliations:** 1 Instituto de Ciencias e Innovación en Medicina, Facultad de Medicina, Clínica Alemana, Universidad del Desarrollo, Av. Plaza 680, Las Condes, Región, Metropolitana Chile; 2 Instituto de Salud Pública de Chile, Subdepartamento Innovación, Desarrollo, Transferencia Tecnológica y ETESA, Departamento Agencia Nacional de Dispositivos Médicos, Innovación y Desarrollo, Santiago, Región Metropolitana Chile

**Keywords:** International migration, Healthy migrant effect, Social determinants of health, Health disparities

## Abstract

**Background:**

During recent decades intraregional migration has increased in Latin America. Chile became one of the main receiving countries and hosted diverse international migrant groups. Evidence have suggested a healthy migrant effect (HME) on health status, but it remains scarce, controversial and needs to be updated. This study performed a comprehensive analysis verifying the existence of HME and its association with social determinants of health (SDH).

**Methods:**

We analyzed data from the Chilean National Socioeconomic Characterization Survey (CASEN, version 2017). Unadjusted prevalence of health status indicators such as negative self-perceived health, chronic morbidity, disability, and activity limitations were described in both international migrants and local population. Adjusted associations between these outcomes and sets of demographics, socioeconomic, access to healthcare, psychosocial and migration-related SDH were tested using multivariate logistic regression in each population. The HME for each health outcome was also tested using multivariate logistic regression and sequentially adjusting for each set of SDH (ref = Chilean).

**Results:**

International migrants had lower unadjusted prevalence of all health indicators compared to Chileans. That is, unadjusted analysis revealed an apparent HME in all health outcomes. Age, unemployment, and health care system affiliation were associated with health outcomes in both populations. Psychosocial determinants were both risk and protective for the analysed health outcomes. After adjustment for each set of SDH, the immigrant health advantage was only significant for chronic morbidity. Being migrant was associated with 39% lower odds of having chronic diseases compared to locals (OR: 0.61; 95% CI: 0.44–0.84; *P* = 0.0003). For all other outcomes, HME disappeared after adjusting by SDH, particularly unemployment, type of health system and psychosocial factors.

**Conclusions:**

Testing the HME in Chile revealed an advantage for chronic morbidities that remained significant after adjustment for SDH. This analysis shed light on health disparities between international migrants and local population in the Latin American region, with special relevance of unemployment, type of health system and psychosocial SDH. It also informed about differential exposures faced during migration process that could dissolve the HME over time. Evidence from this analytical approach is useful for informing health planning and intersectoral solutions from a SDH perspective.

## Background

International migration is a complex process of voluntary or involuntary human mobility [1] that has an influence on health status. Among existing theories related to migration and health, literature has proposed the “healthy migrant effect” (HME) hypothesis. This phenomenon postulates that migrants have on average better health outcomes, empirically observed by lower morbidity and mortality rates when compared to native-born populations. That is, migrants appear to be healthier despite of coming -in many cases- from lower-income developing countries, and despite facing multiple social disadvantages throughout the migration process [2]. One explanatory model has proposed a *positive self-selection * in which those who are healthier and wealthier have a higher chance of moving away from their place of residence [3]. For example, selection applies to those who are younger and those with skills relevant to labor market needs [4]. A second explanation is based on healthy behaviors preserved during the migration process, which could be enhanced with favorable life conditions in the destination country [5]. Moreover, it has been postulated that psychosocial resources like social support and social cohesion may be protective for positive reinforcement of healthy behaviors [6, 7], stress management and disease risk prevention [8].

Approximately 3.6% of the world’s population are international migrants, as estimated in 2020 [9]. In Latin America, one of the main migratory flows throughout the last decades has been intraregional, often known as the south-south migration pattern [10]. Within the region, Chile has experienced a steady increase of international migration fluxes since the early 2000s, with the latest estimations from the National Institute of Statistics reporting 1.462.103 international migrants at the end of 2020 (8% of the total population). The same report indicated that migrant men were slightly overrepresented (50.9%) compared to women, almost half of migrants were aged between 25 and 35 years old and most of international migrants came from countries within the region such as Venezuela (representing about a third of all international migrants in the country), Perú, Haiti, Colombia and Bolivia [11]. Similar to other countries and regions, there is great heterogeneity between the international migrant population and the local population in Chile, as well as within different migrant communities, based on demographic and socioeconomic characteristics [12]. This variability is particularly important, since diverse exposures to demographic, socioeconomic and psychosocial determinants during the migration process might have distinctive influences on health and wellbeing [13, 14]. Furthermore, migration itself has been recognized as a social determinant of health, given the potential effect of certain migration circumstances on health risks [15], which makes the relationship between migration and health a public health priority [13].

In Chile, evidence from population-based studies have reported the probable existence of the HME on unadjusted or crude health indicators, such as disability [16, 17], illness, accidents and chronic health conditions [17]. This has also been reported when analyzing hospital discharges rates in this country, where migrants have had a lower proportion of infectious diseases, metabolic disorders, mental health conditions, and cardiorespiratory diseases [18]. Recent studies have also observed this advantage in the context of emergency consultations of migrants residing in the northern area of the capital of Chile. For instance, migrants reported lower hospitalization rates and lower prevalence of a number of health conditions [19]. Meanwhile, Peruvian mothers living in Santiago have also shown an advantage on perinatal outcomes over native Chilean mothers [20]. Interestingly, some studies have proposed that migrants living in different cities in Chile might have healthier behaviors than locals, as they have reported regular physical activity more often than the Chilean-born, which could in turn promote their integration and increase their psychosocial resources [21].

Among the above-mentioned evidence from the Chilean context, some authors have tested the healthy migrant effect from the perspective of social determinants of health (SDH), defined as “*the conditions in which people are born, grow, work, live and age, and the wider set of forces and systems shaping the conditions of daily life*” [22]. For example, a population-based study showed that the unadjusted advantage of migrants over Chilean population on disability, any health problem and chronic conditions or cancer disappeared after adjusting by socioeconomic determinants like household income and educational level. Similarly, determinants related to the migration process like length of stay in the country also had an influence on migrants health over time, as it seemed that having been in Chile for 10 years or more attenuated and dissolved the healthy migrant effect [17]. Likewise, another study of Chilean hospital discharges proposed that lower discharges rates could be explained by demographic factors (e.g., age) and reduced access to health care [18]. Therefore, positive selection might not apply to all cases. Evidence suggests that diverse exposures during the migration process could influence the health of international migrants, including determinants such as social exclusion, socioeconomic status and poverty [23].

Literature describes SDH that influence the health of international migrants, including: i) economic disadvantage and poor living conditions; ii) the effect of educational level on health literacy and behavioral decisions; iii) public policies and migratory laws acting either as facilitators or limiters [24]; iv) psychosocial determinants that could also promote risk according to context of migration and interactions with the host society; for example, the lack or imbalance of psychosocial resources such as social support and limited social network could have negative impact on health outcomes [8]; v) access to health care often determined by migratory status (regular versus irregular administrative status in the host country) and sociocultural barriers to health care [24]. Noteworthy, previous studies have described that migrant population in Chile are more likely to be uninsured and to report a lower use of healthcare services compared to the local population [25]. International literature has suggested that lower access to healthcare might lead to under-reported existing medical conditions among migrants, raising questions around its influence on HME analysis [26]. Overall, the SDH approach can go beyond merely unadjusted and average comparisons, as it comprehensively explores its influence on the HME.

Currently there is little evidence testing the HME on the health status of international migrants residing in Chile and the Latin American region more generally. Local literature remains inconclusive but recognizes the potential impact of diverse exposures during the migration process. The migrant population in Chile has changed over time in terms of its composition and social determinants, becoming an increasingly heterogeneous group. The evolution of the structure and trends of migration inflows to Chile points out the need of an update analysis of HME under the social determinants of health approach. Since recent evidence does not consider the SDH perspective, conducting this type of analysis would contribute to a more comprehensive understanding of the complex relationship between migration and health, and its implications for public health in Chile and the region. The present study aimed at analyzing the existence of the healthy migrant effect (HME) on self-perceived health, chronic morbidity, disability and activity limitations and its association with different social determinants of health (SDH). This analysis was performed by comparing the health status of international migrants and the Chilean population from a population-representative dataset. In order to test the HME, we investigated the influence of demographic, socioeconomic, access to healthcare, psychosocial and migration-related determinants on such health outcomes. This updated analysis also brings attention to the multidimensional nature of migration in the South American region and the identification of unique determinants of the health among international migrants in Chile from a SDH perspective.

## Methods

Cross-sectional observational study. We conducted a secondary analysis of the National Socioeconomic Characterization Survey (CASEN version 2017). The CASEN survey is regularly applied by the Ministry of Social Development to Chilean households and their residents every two to three years in Chile. Its aim is to describe their socioeconomic situation, multidimensional poverty, and income distribution, as well as to identify updated socioeconomic needs among prioritized underserved groups. It is a voluntary survey that encompasses a structured interview conducted by a trained field interviewer and answered by an adult who provides data on him/herself and all the other household members. This survey is designed with a probabilistic, stratified and multistage sampling that is representative at each national, regional (16 regions), and urban/rural level. It excludes a limited number of hard-to-reach geographical boroughs in the country and institutionalized individuals (people residing in hospitals, prisons, home cares). The total sample of the 2017 CASEN survey comprised 70.947 households and 216.439 residents, representing an estimated total of 16.843.471 Chilean-born inhabitants and 777.407 international migrants (those who reported being born in a different country than Chile, i.e., first generation international migrants) residing in the country at the time of data collection. The CASEN survey dataset is public and free of access upon completing an online form from the Ministry of Social Development Web page [27]. The anonymous dataset can be downloaded after this procedure. This study was part of the Fondecyt Regular project 1,201,461 approved by the Ethics Committee of the Faculty of Medicine of The Universidad del Desarrollo and the Ethics Committee of the Servicio de Salud Metropolitano Sur Oriente (South-East Metropolitan Public Health Service). The study complied with ethical guidelines and regulations according to the principles of the Declaration of Helsinki.

### Health status

Health status was examined using the framework of the health module from the European Statistics of Income and Living Condition (EU-SILC) as a reference. The instrument contains 3 different variables with its corresponding concepts [28]. These concepts were used to create new variables from the questions available in the CASEN survey.

*Negative Self-perceived health (NSPH):* the new variable was created based on the question “from 1 to 7 how would you rate your current health status”. According to previous literature the seven-grade scale could be interpreted as 1 very poor health to 7 excellent health that could not be improved [29]. Like previous studies [30], scores ranging 4–7 represented positive health and scores ranging 1–3 represented negative health. This study focused on negative self-perceived health as an indicator, in order to maintain consistency with the other negative health indicators included in the analysis.

*Chronic morbidity (CM)*: based on the question “have you been receiving medical treatment for the past 12 months?”. Dichotomized as yes or no according to the presence of hypertension/dental Emergency, diabetes, depression, acute myocardial infarction, cataracts, chronic obstructive pulmonary disease, leukemia, bronchial asthma, cancer (gastric, cervical uterine, breast, testicular, prostate, colorectal), preventive cholecystectomy, chronic kidney failure, ischemic brain accident, bipolar disorder, lupus or another chronic condition.

*Disability (DIS)*: although the EU-SILC framework does not separate disability from the activity limitation variable, the CASEN survey includes the following question focused on disability [31], from whom the new variable was created: “Do you have any of the following permanent conditions?” Dichotomized as yes or no according to the presence of one or more physical/speaking/psychiatric/mental/hearing/visual conditions.

*Activity limitations (AL)*: The variable was created using all types of daily living activities limitations asked by CASEN. “How much difficulty do you have for...?”. This question was restricted to population over 6 years. Dichotomized as yes or no according to the presence of mild, moderate, severe, or extreme difficulty for one or more activities (eating, showering, displacing, using the bathroom, lying down or getting out of bed/ getting dressed).

### Social determinants of health

*Demographic determinants*: age as a continuous and categorical variable (< 6 years, 6–14, 15–29, 30–44, 45–64 and > 64 years). Sex (male, female). Ethnicity for those belonging to or being descendant of minority groups in Chile (yes, no), marital status (single, married/cohabitant, separated/divorced/annulled, widow), area (urban, rural).

*Socioeconomic determinants*: educational level according to the highest level achieved or current level of the household informant (categorized as university or higher, technical, high school, primary, none) according to the adjusted and standardized Chilean educational system, in which the gross categories share similarities with other systems within the region. This categorization has been used in previous Chilean research performing demographic analysis of both local and migrant population [16, 17]. Household income categorized in five quintiles of equal size sorted in ascending order according to the autonomous per capita household income (I: 273.414 Chilean pesos equivalent to 414 US dollars; II: 486.332 Chilean pesos equivalent to 736 US dollars; III: 687.569 Chilean pesos equivalent to 1040 US dollars; IV: 951.021 Chilean pesos equivalent to 1438 US dollars; V: 2.331.479 Chilean pesos equivalent to 3.526 US dollars). Occupation defined by the occupational activity of the household informant. The variable was created from questions related to current job/occasional job/on work leave/searching for a job/ attending an educational institution (categorized as unemployed, is not studying, studying, employed, and studying and working).

*Access to health care*: The variable affiliation to the health care system was used as a proxy of access and created from the question “Which health insurance system do you use?”. Further categorized as none, public health system affiliation, private health system affiliation, other.

*Psychosocial factors*: These factors refer to characteristics that could have a psychological and/or social impact on an individual, involving social-level and individual-level processes [32]. Among psychosocial factors, there are protective resources in the social environment such as social support, defined as the perception of value, affection and care from others and social capital related to reciprocal interactions based on trust [32, 33]. The social support variable was created from available questions that were mainly related to instrumental social support networks; dichotomized yes or no according to the presence of one or more supportive behaviors from someone at home and outside. The social capital variable was created from a question focusing on belonging and participation in diverse organizations or organized groups over the last 12 months. It was dichotomized as yes or no according to the participation in one or more of these groups.

*Migration-related factors*: the” country of origin” variable was created as a categorical variable based on the question “When you were born, what country did your mother live in?”. The categories were selected according to the intraregional pattern reported in migratory statistics [11] (Venezuela, Perú, Haiti, Colombia, Bolivia, Argentina, Ecuador, other countries in South America and other). The “length of stay or time of residence in the country” variable was created based on the year period in which the migrant arrived and categorized (2015 or later, 2010–2014, 2005–2009, 2000–2004, 1999 or before).

### Statistical analysis

Health status outcomes were analyzed descriptively for international migrants and the Chilean born population separately. Unadjusted (or crude) prevalence of selected health outcomes was presented as proportions and then stratified by demographic, socioeconomic, access to health care and migration-related determinants. The Pearson’s chi-square test was used to test independence between migration status (migrant versus Chilean) and health status outcomes. Multivariate logistic regression was used to estimate the probability (odds ratio, OR) of reporting these health outcomes crude and adjusted by each set of SDH in international migrants and local population separately. The association between migration-related factors and health outcomes was explored with multivariate logistic regression adjusted by sex and age. Then, the healthy migrant effect (HME) was examined using multivariate logistic regression sequentially adjusted for SDH, where NSPH, CM, DIS and AL were dependent variables and migratory status was the main independent variable (reference: Chilean born). The Hosmer-Lemeshow goodness of fit test was used as post-estimation after logistic regression. Data analyses were performed with STATA 14 software (Stata Corp) and weighted according to the survey’s sampling design. Significance was set at 0.05 with 95% confidence interval (95% CI).

## Results

The total sample represented 16.843.471 Chilean-born individuals and 777.407 international migrants (4.4% of the total population in Chile based on the CASEN 2017 analysis). The majority of the sample were women (52.5% Chileans and 51.4% migrants). Regarding the age distribution, over 50% of the migrant population were aged 15 to 44 years (34.6% were 15–29 years and 36.1% were 30–44 years old). Meanwhile, 22.9% of the local population were 15–29 years old and 17.9% ranged between 30 and 44 years old. Furthermore, 49.7% of Chileans and 44.9% of migrants were single. The educational level differed across these populations, 0.6% of migrants did not have formal education compared to 2.4% of the native born. In addition, 35.8% of migrants had higher education, whereas 17.4% of Chileans did.

### Unadjusted (crude) prevalence of health outcomes

International migrants had lower crude prevalence of NSPH (3.97% vs 5.91%), CM (9.55% vs. 25.97%), DIS (14.63% vs. 23.89%) and AL (5.56 vs. 11.52%). After stratifying by SDH, both groups showed higher prevalence of health status outcomes among females, people over 64 years, widows, the unemployed and those affiliated to the public health system. The outcomes differed by geographical area, for example prevalence of CM and DIS were higher among local and migrant population living in rural areas. In contrast, negative self-perceived health and AL were higher among Chileans living in rural areas but lower among migrants. When stratifying by socioeconomic determinants, we observed a clear social gradient in self-perceived health across income quintiles of both groups, migrants and the Chilean-born. Among migrants, higher prevalence of NSPH was observed in those who were uninsured and those with only primary level education. However, CM was higher in migrants with the highest education level and those with private health system affiliation. Conversely, DIS and AL, was higher in those who were affiliated to the public health system (Tables 1, 2 and 3). Furthermore, migrants from Peru showed the highest prevalence of NSPH (6.43%), disability (7.69%) and AL (4.15%) whereas those from Ecuador had the higher percentage of CM (23.37%). Meanwhile, migrants who had arrived in 2015 or later showed higher rates of negative health perception (4.5%), but those who expended more than 20 years had higher rates of CM (31.08%), DIS (16.58%) and activity AL (7.34%) (Table 4).

**Table 1:** Unadjusted global and stratified prevalence of health outcomes by SDH factors in immigrant population (*n* = 777.407)

Social determinant of health	Negative self-perceived health		Chronic morbidity		Disability	
	***n*** = 30.601		***n*** = 74.216		***n*** = 43.236	
	%	95% CI	%	95% CI	%	95% CI
	3.97%	[2.8–5.7%]	9.55%	[8.3–10.9%]	14.63%	[13.1–16.3%]
**Sex**						
Female	4.67%	[2.7–8.0%]	10.34%^****^	[8.9–12.0%]	7.07%^***^	[5.0–10.0%]
Male	3.22%^**^	[2.4–4.4%]	8.92%^****^	[6.6–12.0%]	4.01%^****^	[3.1–5.2%]
**Age categories**						
< 6	1.66%^****^	[0.5–5.3%]	5.00%^****^	[2.6–9.5%]	6.45%^****^	[3.4–11.8%]
6–14 years	3.78%^****^	[2.1–6.6%]	3.21%^****^	[1.6–6.3%]	6.02%^****^	[3.8–9.3%]
15–29 years	2.90%^****^	[1.8–4.6%]	5.28%^****^	[2.5–10.7%]	2.98%^****^	[2.0–4.5%]
30–44 years	3.93%^****^	[1.6–9.5%]	7.40%^****^	[6.1–9.0%]	5.53%^****^	[3.0–10.0%]
45–64 years	5.40%^****^	[4.0–7.2%]	23.55%^****^	[19.4–28.2%]	6.29%^****^	[4.8–8.2%]
65 years or more	13.33%^****^	[8.9–19.5%]	49.88%^****^	[41.9–57.9%]	29.41%^****^	[22.3–37.6%]
**Ethnicity**						
Yes	5.67%	[3.6–8.8%]	10.44%^****^	[7.5–14.5%]	7.40%^*^	[5.2–10.4%]
No	3.92%^*^	[2.7–5.7%]	9.63%^****^	[8.4–11.1%]	5.53%^****^	[4.2–7.3%]
**Marital Status**						
Single	4.21%	[2.1–8.4%]	7.71%^**^	[5.4–11.0%]	6.05%	[3.7–9.6%]
Married/cohabitant	3.02%^****^	[2.2–4.1%]	9.35%^****^	[7.9–11.1%]	4.39%^****^	[3.6–5.4%]
Separated/divorced/annulled	7.55%	[3.8–14.6%]	22.55%^****^	[15.8–31.1%]	6.16%^***^	[3.4–10.9%]
Widow	19.81%	[12.1–30.8%]	46.18%^****^	[35.3–57.4%]	32.13%	[22.4–43.7%]
**Area**						
Urban	4.02%	[2.8–5.8%]	9.51%^****^	[8.2–11.0%]	5.56%^****^	[4.2–7.3%]
Rural	2.40%^**^	[1.2–4.9%]	13.76%^****^	[9.7–19.1%]	6.27%^***^	[4.2–9.3%]
**Educational level**						
None	3.65%	[1.8–7.3%]	5.60%^****^	[3.2–9.6%]	8.42%^*^	[5.2–13.3%]
University	2.90%	[1.7–4.9%]	13.96%^*^	[9.9–19.4%]	3.80%^**^	[2.7–5.4%]
Technical	1.68%^**^	[1.0–3.0%]	7.52%^****^	[5.2–10.8%]	3.56%^**^	[2.0–6.1%]
High School	4.73%	[2.4–9.3%]	7.77%^****^	[6.5–9.3%]	5.91%^*^	[3.5–9.9%]
Primary	5.67%^**^	[4.2–7.7%]	9.95%^****^	[7.7–12.8%]	8.25%^****^	[6.3–10.7%]
**Income quintile**						
I	4.71%^*^	[3.0–7.4%]	8.73%^****^	[6.5–11.7%]	5.63%^****^	[3.7–8.4%]
II	6.01%	[4.3–8.4%]	8.68%^****^	[6.9–10.9%]	7.68%^**^	[5.2–11.2%]
III	5.80%	[1.9–16.8%]	8.29%^****^	[6.3–10.8%]	9.40%	[4.7–18.0%]
IV	1.91%^****^	[1.3–3.0%]	7.73%^****^	[6.2–9.6%]	3.93%^****^	[2.8–5.5%]
V	3.30%	[2.0–5.3%]	14.10%^****^	[10.1–19.3%]	3.13%^****^	[2.1–4.7%]
**Occupation**						
Does not study	0.91%	[0.2–3.6%]	2.73%	[1.0–7.6%]	4.12%	[1.5–11.0%]
Unemployed	9.41%	[4.8–17.8%]	17.46%^****^	[14.1–21.4%]	13.05%^**^	[8.1–20.3%]
Studying	3.85%	[1.1–12.2%]	8.53%	[5.2–13.8%]	4.33%	[2.5–7.4%]
Employed	2.59%^**^	[1.9–3.5%]	7.37%^****^	[6.0–9.0%]	3.38%^****^	[2.7–4.3%]
Studying or/and employed	0.54%^*^	[0.1–2.9%]	45.35%^**^	[13.9–81.0%]	1.92%	[0.5–6.6%]
**Access to healthcare**						
None	2.48%^**^	[1.5–4.2%]	3.78%^****^	[2.5–5.6%]	4.70%^*^	[2.8–7.8%]
Public health system affiliation	4.36%	[2.7–7.0%]	9.15%^****^	[7.8–10.8%]	6.48%^****^	[4.7–8.9%]
Private health system affiliation	4.10%	[2.1–7.9%]	18.78%	[11.1–29.9%]	2.95%^****^	[1.9–4.5%]
Others	2.55%	[1.0–6.2%]	13.67%^**^	[7.6–23.3%]	3.93%^**^	[1.7–8.7%]
**Social Support**						
Yes	4.33%^***^	[3.2–5.9%]	13.54%^****^	[10.7–17.1%]	4.30%^****^	[3.3–5.6%]
No	30.13%	[6.6–72.5%]	3.54%^****^	[1.4–8.7%]	30.54%	[6.8–72.7%]
**Social capital**						
Yes	3.93%^**^	[2.7–5.7%]	15.42%^****^	[12.5–18.9%]	6.03%^****^	[4.6–7.9%]
No	4.05%^*^	[2.6–6.3%]	9.05%^****^	[7.4–11.0%]	5.44%^****^	[3.9–7.6%]

^*^p value < 0.05; ^**^p value < 0.01; ^***^p value < 0.001; ^****^p value < 0.0001 when comparing the same category between the Chilean-born and the immigrant populations (Chi-square test). *CI* confidence interval

**Table 2:** Unadjusted global and stratified prevalence of health outcomes by SDH factors in Chilean born population (*n* = 16.843.471)

Social determinant of health	Negative self-perceived health		Chronic morbidity		Disability	
	***n*** = 985.235		***n*** = 4.374.959		***n*** = 1.939.571	
	%	95% CI	%	95% CI	%	95% CI
	5.91%	[5.7–6.1%]	25.97%	[25.6–26.4%]	23.89%	[23.6–24.2%]
Sex						
Female	6.61%	[6.4–6.9%]	30.55%^****^	[30.0–31.1%]	12.59%^***^	[12.2–13.0%]
Male	5.14%^**^	[4.9–5.4%]	21.47%^****^	[21.1–21.9%]	10.43%^****^	[10.1–10.7%]
Age categories						
< 6	2.81%^***^	[2.5–3.2%]	7.95%^***^	[7.2–8.7%]	5.10%^***^	[5.4–6.4%]
6–14 years	2.61%^***^	[2.3–2.9%]	8.56%^***^	[8.1–9.1%]	5.10%^***^	[4.6–5.6%]
15–29 years	2.48%^***^	[2.3–2.7%]	8.85%^***^	[8.4–9.3%]	5.87%^***^	[5.4–6.4%]
30–44 years	4.25%^***^	[3.9–4.6%]	16.29%^***^	[15.7–17.0%]	5.87%^***^	[5.4–6.4%]
45–64 years	7.98%^***^	[7.7–8.3%]	39.44%^***^	[38.8–40.1%]	8.93%^***^	[8.6–9.2%]
65 years or more	14.23%^***^	[13.7–14.8%]	67.80%^***^	[67.0–68.6%]	32.04%^***^	[31.1–32.9%]
Ethnicity						
Yes	5.69%	[5.2–6.2%]	21.36%^****^	[20.5–22.2%]	11.24%^*^	[10.6–11.9%]
No	5.93%^*^	[5.7–6.1%]	26.78%^****^	[26.4–27.2%]	11.61%^****^	[11.3–11.9%]
Marital Status						
Single	3.77%	[3.6–4.0%]	13.57%^**^	[13.1–14.0%]	8.17%	[7.9–8.5%]
Married/cohabitant	7.15%^****^	[6.9–7.4%]	35.04%^****^	[34.4–35.7%]	12.43%^****^	[12.0–12.9%]
Separated/divorced/annuled	8.54%	[7.9–9.2%]	40.18%^****^	[39.0–41.3%]	15.12%^***^	[14.3–16.0%]
Widow	14.54%	[13.7–15.4%]	67.53%^****^	[66.4–68.7%]	35.48%	[34.2–36.8%]
Area						
Urban	5.80%	[5.6–6.0%]	25.93%^****^	[25.5–26.4%]	11.45%^****^	[11.1–11.8%]
Rural	6.67%^**^	[6.3–7.1%]	28.31%^****^	[27.4–29.2%]	12.34%^***^	[11.7–13.0%]
Educational level						
None	6.32%	[5.9–6.8%]	18.08%^****^	[17.2–19.0%]	13.89%^*^	[13.2–14.6%]
University	2.92%	[2.7–3.2%]	19.89%^*^	[19.2–20.6%]	6.78%^**^	[6.3–7.3%]
Technical	3.71%^**^	[3.3–4.2%]	21.58%^****^	[20.5–22.7%]	7.57%^**^	[6.9–8.3%]
High School	5.71%	[5.5–6.0%]	26.68%^****^	[26.1–27.2%]	10.60%^*^	[10.3–11.0%]
Primary	8.30%^**^	[8.0–8.6%]	33.48%^****^	[32.8–34.1%]	15.74%^****^	[15.2–16.3%]
Income quintile						
I	7.89%^*^	[7.5–8.3%]	27.31%^****^	[26.6–28.0%]	14.24%^****^	[13.7–14.8%]
II	6.60%	[6.3–7.0%]	26.08%^****^	[25.4–26.7%]	12.34%^**^	[11.8–12.9%]
III	6.08%	[5.7–6.5%]	26.58%^****^	[25.8–27.4%]	11.92%	[11.4–12.5%]
IV	5.02%^****^	[4.7–5.4%]	26.45%^****^	[25.7–27.2%]	10.20%^****^	[9.7–10.7%]
V	2.99%	[2.7–3.3%]	24.33%^****^	[23.4–25.3%]	7.90%^****^	[7.3–8.5%]
Occupation						
Does not study	2.91%	[2.4–3.5%]	6.59%	[5.8–7.5%]	5.03%	[4.4–5.8%]
Unemployed	11.88%	[11.5–12.3%]	48.73%^****^	[48.1–49.4%]	23.46%^**^	[22.9–24.1%]
Studying	2.10%	[1.9–2.4%]	9.49%	[8.9–10.2%]	5.51%	[5.1–6.0%]
Employed	4.20%^**^	[4.0–4.4%]	23.54%^****^	[23.1–24.0%]	7.61%^****^	[7.3–7.9%]
Studying or/and employed	2.94%^*^	[2.2–3.9%]	10.76%^**^	[9.5–12.2%]	5.71%	[4.6–7.1%]
Access to healthcare						
None	4.98%^**^	[4.1–6.1%]	13.63%^****^	[12.3–15.1%]	8.01%^*^	[6.9–9.2%]
Public health system affiliation	6.54%	[6.3–6.7%]	27.77%^****^	[27.3–28.2%]	12.68%^****^	[12.4–13.0%]
Private health system affiliation	2.86%	[2.5–3.2%]	20.68%	[19.7–21.7%]	6.34%^****^	[5.8–6.9%]
Others	5.66%	[4.9–6.6%]	28.49%^**^	[26.4–30.7%]	11.22%^**^	[9.9–12.6%]
Social Support						
Yes	7.54%^***^	[7.3–7.8%]	40.40%^****^	[39.8–41.0%]	15.00%^****^	[14.5–15.5%]
No	14.06%	[11.8–16.6%]	40.92%^****^	[36.7–45.3%]	20.26%	[17.4–23.5%]
Social capital						
Yes	6.36%^**^	[6.1–6.7%]	36.23%^****^	[35.6–36.9%]	13.71%^****^	[13.2–14.2%]
No	6.41%^*^	[6.2–6.6%]	26.18%^****^	[25.7–26.6%]	12.05%^****^	[11.7–12.4%]

^*^p value < 0.05; ^**^p value < 0.01; ^***^p value < 0.001; ^****^p value < 0.0001 when comparing the same category between the Chilean-born and the immigrant populations (Chi-square test). *CI* confidence interval

**Table 3:** Unadjusted global and stratified prevalence of activity limitations by SDH in immigrant (*n* = 746.600) and Chilean born population (*n* = 15.538.162), aged 6 years or more

Social determinants of health	Activity limitations			
	Chilean born population		Migrant population	
	***n*** = 812.277		***n*** = 17.846	
	%	95% CI	%	95% CI
	11.52%	[11.2–11.8%]	5.56%	[4.3–7.2%]
Sex				
Female	6.18%	[6.0–6.4%]	3.39%	[1.6–7.1%]
Male	4.16%^****^	[4.0–4.4%]	1.33%^****^	[0.9–2.1%]
Age categories				
< 6				
6–14 years	4.57%^****^	[4.2–5.0%]	4.46%^****^	[1.9–10.4%]
15–29 years	0.81%^****^	[0.7–0.9%]	0.34%^****^	[0.2–0.8%]
30–44 years	1.27%^****^	[1.1–1.5%]	2.45%^****^	[0.6–9.5%]
45–64 years	4.59%^****^	[4.3–4.9%]	1.75%^****^	[0.1–2.8%]
65 years or more	19.01%^****^	[18.4–19.7%]	21.14%^****^	[15.1–28.8%]
Ethnicity				
Yes	4.68%	[4.3–5.1%]	4.79%	[3.1–7.4%]
No	5.29%^**^	[5.1–5.5%]	2.32%^**^	[1.3–4.2%]
Marital Status				
Single	3.50%	[3.3–3.7%]	3.16%	[1.2–8.2%]
Married/cohabitant	4.65%^****^	[4.4–4.9%]	1.12%^****^	[0.7–1.7%]
Separated/divorced/annulled	6.34%^****^	[5.8–7.0%]	1.17%^****^	[0.5–2.9%]
Widow	23.99%	[22.9–25.1%]	29.22%	[19.9–40.7%]
Area				
Urban	5.15%^**^	[5.0–5.3%]	2.40%^**^	[1.3–4.3%]
Rural	5.75%^**^	[5.4–6.1%]	2.21%^**^	[1.2–4.0%]
Educational level				
None	24.50^*^	[23.0–26.1%]	9.33%^*^	[3.0–25.3%]
University	1.73%^*^	[1.5–2.0%]	0.95%^*^	[0.6–1.6%]
Technical	1.66%^**^	[1.4–2.0%]	0.49%^**^	[0.2–1.2%]
High School	3.78%	[3.6–4.0%]	2.85%	[0.9–8.3%]
Primary	8.19%^**^	[7.9–8.5%]	4.15%^**^	[2.6–6.6%]
Income quintile				
I	7.38%^****^	[7.0–7.8%]	2.86%^****^	[1.8–4.6%]
II	5.76%^****^	[5.5–6.1%]	1.71%^****^	[1.1–2.6%]
III	5.15%	[4.8–5.5%]	5.17%	[1.4–17.7%]
IV	4.16%^*^	[3.9–4.5%]	1.75%^*^	[0.9–3.6%]
V	2.99%^***^	[2.7–3.3%]	1.38%^***^	[0.9–2.2%]
Occupation				
Does not study	33.81%^****^	[21.8–48.3%]	1.77%^****^	[0.2–14.0%]
Unemployed	12.33%	[11.9–12.7%]	7.69%	[3.4–16.5%]
Studying	0.62%	[0.5–0.8%]	0.95%	[0.4–2.5%]
Employed	1.61%^****^	[1.5–1.8%]	0.61%^****^	[0.4–1.1%]
Studying and work	0.29%	[0.1–0.6%]	0.93%	[0.2–5.6%]
Access to healthcare				
None	2.51%	[1.9–3.2%]	1.58%	[0.7–3.7%]
Public health system affiliation	5.92%^*^	[5.7–6.1%]	2.75%^*^	[1.3–5.8%]
Private health system affiliation	1.93%	[1.7–2.2%]	1.47%	[0.8–2.7%]
Others	5.84%	[4.9–6.9%]	2.71%	[1.0–7.0%]
Social support				
Yes	6.48%^****^	[6.2–6.8%]	1.62%^****^	[1.0–2.5%]
No	8.18%	[6.6–10.1%]	29.01%	[5.8–73.0%]
Social capital				
Yes	5.42%^****^	[5.2–5.7%]	2.06%^****^	[1.3–3.2%]
No	5.19%^*^	[5.0–5.4%]	2.30%^*^	[1.1–4.8%]

^*^p value < 0.05; ^**^p value < 0.01; ^***^p value < 0.001; ^****^p value < 0.0001 when comparing the same category between the Chilean-born and the immigrant populations (Chi-square test). *CI* confidence interval

**Table 4:** Crude and stratified prevalence of health outcomes by migration-related factors in immigrant population

	Negative self-perceived health		Chronic morbidity		Disability		Activity limitations	
	%	95% CI	%	95% CI	%	95% CI	%	95% CI
**Country of Origin**								
Venezuela	1.93%	[0.94–3.90%]	7.90%	[3.97–15.11%]	4.36%	[2.57–7.33%]	0.96%	[0.44–2.07%]
Peru	6.43%	[2.71–14.50%]	8.65%	[6.94–10.74%]	7.69%	[3.76–15.08%]	4.15%	[1.04–15.21%]
Haiti	4.67%	[2.60–8.25%]	1.79%	[0.79–3.99%]	2.94%	[1.65–5.17%]	0.58%	[0.20–1.70%]
Colombia	2.75%	[1.37–5.47%]	6.22%	[4.14–9.25%]	4.23%	[2.50–7.06%]	1.91%	[0.69–5.18%]
Bolivia	2.95%	[1.89–4.59%]	7.11%	[5.22–9.63%]	5.85%	[4.28–7.93%]	3.15%	[2.12–4.67%]
Argentina	2.36%	[1.22–4.52%]	20.49%	[16.24–25.50%]	7.21%	[5.04–10.22%]	2.27%	[1.28–3.99%]
Ecuador	4.48%	[2.41–8.17%]	23.37%	[14.00–36.37%]	4.47%	[2.55–7.71%]	1.37%	[0.43–4.24%]
Other countries in South America	4.61%	[1.95–10.49%]	14.46%	[9.40–21.59%]	4.06%	[1.59–10.01%]	1.53%	[0.46–4.93%]
Others	5.79%	[3.81–8.71%]	18.52%	[14.63–23.17%]	7.89%	[5.80–10.65%]	4.32%	[2.96–6.27%]
**Time of residence**								
2015 o later	4.56%	[2.71–7.58%]	8.04%	[3.47–17.54%]	3.00%	[2.11–4.23%]	0.23%	[0.03–1.62%]
2010–2014	3.71%	[1.14–11.37%]	6.84%	[3.40–13.27%]	3.12%	[1.29–7.34%]	2.48%	[1.69–3.63%]
2005–2009	1.11%	[0.33–3.67%]	4.46%	[1.16–15.67%]	8.07%	[3.23–18.76%]	0.29%	[0.04–2.13%]
2000–2004	0.73%	[0.16–3.28%]	7.99%	[2.57–22.18%]	3.65%	[0.58–19.76%]	0.23%	[0.03–1.72%]
1999 or before	2.68%	[0.86–7.99%]	31.08%	[21.69–42.34%]	16.58%	[10.02–26.19%]	7.34%	[3.38–15.23%]
doesn’t know	4.00%	[2.11–6.70%]	13.73%	[10.84–17.23%]	6.12%	[4.51–8.24%]	3.47%	[2.39–5.03%]

*CI* confidence interval

### SDH associated with health outcomes

Logistic regression models for NSPH, CM and DIS adjusted by different set of SDH in migrant population are presented in Table 5. Models for Activity Limitations in both populations are presented in Table 6. Age was associated with all health outcomes in both populations. Among international migrants, after adjusting for demographic variables, the odds of having NSPH was 7.44 times higher in those unemployed (OR: 7.44; 95% CI: 1.05–52.61). CM was associated with affiliation to the health system, particularly affiliation to the private health system (OR 4.99; 95% CI: 2.70–9.25), whereas the risk of CM was also associated with having social support (OR: 3.29; 95% CI: 1.29–8.40) and social capital (OR: 1.84; 95% CI:1.23–2.75). Conversely, social support was associated with reduced odds of DIS (OR: 0.23; 95% CI: 0.09–0.60). Moreover, other variables were associated with both reduced and higher odds, for example having social support reduced by 77% the odds of NSPH (OR: 0.23; 95% CI: 0.10–0.55) and AL (OR: 0.13; 95% CI: 0.05–0.35) but increased the odds for CM (OR: 3.29; 95% CI: 1.29–8.40). Likewise, being married/cohabitating was associated with less chances of DIS (OR: 0.50; 95% CI: 0.29–0.87) and AL (OR: 0.24; 95% CI: 0.09–0.62). Regarding migration-related factors (Table 7.), those from Haiti had higher odds of NSPH (OR: 4.67; 95% CI: 1.31–16.66) and DIS (OR: 2.88; 95% CI: 0.15–7.19), while those from Argentina showed higher risk of CM (OR: 1.42; 95% CI: 0.59–3.42). Staying over 20 years in Chile was associated with 11.04 times more chances of DIS (OR: 11.04; 95% CI: 3.65–33.40).

**Table 5:** Logistic regression models of health outcomes by SDH in immigrant population

	Self-perceived bad health			Chronic morbidity			Disability		
	OR	95% CI	*P* value	OR	95% CI	*P* value	OR	95% CI	*P* value
**Demographic**									
Age	1.02****	**[1.01–1.03]**	**< 0.000**	1.06****	**[1.05–1.07]**	**< 0.000**	1.03****	**[1.02–1.04]**	**< 0.000**
Sex (ref = male)	1.33	[0.67–2.66]	0.410	1.01	[0.61–1.65]	0.983	1.65*	**[1.05–2.60]**	**0.029**
Ethnicity: (ref = no ethnicity)	1.62	[0.89–2.95]	0.114	0.85	[0.52–1.41]	0.532	1.29	[0.82–2.04]	0.267
Marital status (ref = single)									
Married/Cohabitant	0.54	[0.23–1.29]	0.167	0.65	[0.41–1.05]	0.076	0.50**	**[0.29–0.87]**	**0.014**
Separated/divorced/annuled	1.07	[0.32–3.57]	0.917	1.01	[0.57–1.80]	0.965	0.49	[0.22–1.10]	0.081
Widow	1.66	[0.42–6.57]	0.473	0.67	[0.37–1.19]	0.169	1.50	[0.60–3.77]	0.387
Zone (ref = urban)	0.48**	**[0.27–0.83]**	**0.009**	1.13	[0.67–1.93]	0.628	0.96	[0.66–1.40]	0.848
GOF test			< 0.000			0.033			0.129
**Socioeconomic**									
Educational level (ref = none)									
University	0.72	[0.22–2.39]	0.596	1.56	[0.58–4.18]	0.380	0.33*	**[0.14–0.82]**	**0.017**
Technical	0.39	[0.12–1.31]	0.128	1.04	[0.41–2.63]	0.933	0.29**	**[0.12 - 0.75]**	**0.011**
High School	0.92	[0.29–2.97]	0.891	1.14	[0.46–2.79]	0.777	0.42*	**[0.19–0.97]**	**0.042**
Primary	1.12	[0.38–3.30]	0.836	1.37	[0.57–3.29]	0.484	0.48	[0.22 - 1.03]	0.061
Income quintile (ref = I lower income level)									
II	1.43	[0.75–2.73]	0.273	1.09	[0.66–1.78]	0.742	1.49	[0.76 - 2.92]	0.245
III	1.43	[0.36–5.67]	0.609	0.92	[0.56–1.52]	0.758	2.44	[0.85 - 7.00]	0.097
IV	0.54	[0.27–1.10]	0.090	0.82	[0.50–1.36]	0.446	0.99	[0.47–2.05]	0.969
V	0.86	[0.35–2.14]	0.753	1.16	[0.68–1.97]	0.588	0.81	[0.36 - 1.80]	0.602
Occupation (ref = does not study)									
Unemployed	7.44*	**[1.05–52.61]**	**0.044**	0.54	[0.13–2.24]	0.400	2.61	[0.52 - 13.17]	0.242
Studying	4.01	[0.52–31.04]	0.183	1.28	[0.32–5.15]	0.614	1.66	[0.38 - 7.29]	0.503
Employed	2.98	[0.51–17.22]	0.223	0.35	[0.10–1.30]	0.118	1.03	[0.26 - 4.08]	0.966
Studying and work	0.73	[0.07–7.47]	0.793	5.30	[0.57–49.68]	0.144	0.91	[0.15 - 5.44]	0.919
GOF test			0.084			0.536			0.220
**Access to healthcare (ref = none)**									
Public health system affiliation	2.02	[0.93–4.42]	0.076	2.94****	**[1.82–4.73]**	**< 0.000**	1.41	[0.68–2.96]	0.357
Private health system affiliation	3.40**	**[1.30–8.86]**	**0.012**	4.99****	**[2.70–9.25]**	**< 0.000**	0.77	[0.34 - 1.72]	0.521
Other	1.11	[0.39–3.10]	0.849	2.99**	**[1.29–6.91]**	**0.011**	0.74	[0.26 - 2.17]	0.593
Doesn’t know	1.28	[0.50–3.26]	0.602	0.98	[0.38–2.57]	0.973	0.73	[0.26 - 2.11]	0.567
GOF test			0.574			0.000			0.445
**Psychosocial**									
Social support (ref = no)	0.23***	**[0.10–0.55]**	**0.001**	3.29**	**[1.29–8.40]**	**0.013**	0.23**	**[0.09 - 0.60]**	**0.003**
Social capital (ref = no)	0.88	[0.51–1.53]	0.651	1.84*	**[1.23–2.75]**	**0.030**	1.09	[0.59–2.01]	0.774
GOF test			0.014			< 0.000			0.747

*CI* confidence interval; ^*^p value < 0.05; ^**^p value < 0.01; ^***^p value < 0.001; ^****^p value < 0.0001

**Table 6:** Logistic regression models of activity limitations by SDH in immigrant and Chilean born populations

	Activity limitations					
	Immigrant			Chilean born		
	OR	95% CI	P value	OR	95% CI	P value
**Demographic**						
Age	1.04^***^	**[1.02–1.07]**	**0.001**	1.06^****^	**[1.05–1.06]**	**< 0.000**
Sex (ref = male)	2.13	[0.77 - 5.90]	0.146	1.18^****^	**[1.12–1.24]**	**< 0.000**
Ethnicity: (ref = no ethnicity)	2.25^*^	**[1.13–4.50]**	**0.021**	1.17^***^	**[1.07–1.28]**	**0.001**
Marital status (ref = single)						
Married/Cohabitant	0.24^**^	**[0.09–0.62]**	**0.003**	0.42^****^	**[0.39–0.45]**	**< 0.000**
Separated/divorced/annuled	0.15^**^	**[0.04 - 0.55]**	**0.004**	0.54^****^	**[0.48–0.60]**	**< 0.000**
Widow	1.67	[0.46 - 6.01]	0.434	0.89^*^	**[0.81–0.98]**	**0.014**
Zone (ref = urban)	0.69	[0.37–1.28]	0.239	1.04	[0.96–1.12]	0.384
GOF test			< 0.000			< 0.000
**Socioeconomic**						
Educational level (ref = none)						
University	0.36	[0.10 - 1.27]	0.112	0.22^****^	**[0.18–0.27]**	**< 0.000**
Technical	0.20^*^	**[0.43 - 0.90]**	**0.037**	0.22	[0.18–0.27]	0.057
High School	1.03	[0.31 - 3.43]	0.958	0.29	[0.26–0.34]	0.235
Primary	0.67	[0.20 - 2.30]	0.527	0.38	[0.33–0.43]	0.604
Income quintile (ref = I lower income level)						
II	0.71	[0.31 - 1.65]	0.434	0.93	[0.86–1.01]	0.083
III	4.23	[0.89 - 19.98]	0.069	0.85^****^	**[0.77–0.93]**	**< 0.000**
IV	0.98	[0.31–3.08]	0.975	0.79^****^	**[0.71–0.88]**	**< 0.000**
V	1.12	[0.38 - 3.33]	0.840	0.73^****^	**[0.63–0.84]**	**< 0.000**
Occupation (ref = none)						
Studying or/and employed	1.66	[0.38 - 7.29]	0.503	0.01^****^	**[0.00–0.02]**	**< 0.000**
GOF test			0.220			< 0.000
**Access to healthcare (ref = none)**						
Public health system affiliation	1.32	[0.34–5.18]	0.690	1.33	[0.99–1.79]	0.058
Private health system affiliation	1.21	[0.32 - 4.67]	0.777	0.95	[0.68–1.31]	0.740
Other	0.55	[0.08 - 3.55]	0.527	1.21	[0.85–1.74]	0.292
Doesn’t know	1.45	[0.38 - 5.54]	0.584	1.15	[0.80–1.68]	0.450
GOF test			0.445			< 0.000
**Psychosocial**						
Social support (ref = no)	0.13^****^	**[0.05–0.35]**	**< 0.000**	1.06	[0.83–1.35]	0.648
Social capital (ref = no)	0.72	[0.29–1.81]	0.484	0.83^****^	**[0.76–0.90]**	**< 0.000**
GOF test			0.747			< 0.000

*CI* confidence Interval; ^*^p value < 0.05; ^**^p value < 0.01; ^***^p value < 0.001; ^****^p value < 0.0001

**Table 7:** Logistic regression models of health outcomes by migration-related factors in immigrant population

	Negative Self-perceived health			Chronic morbidity			Disability		
	OR	95% CI	*P* value	OR	95%CI	*P* value	OR	95% CI	*P* value
*sex + age +*									
**Country of Origin (ref = Peru)**									
Venezuela	2.57	[0.29–22.78]	0.395	1.91	[0.43–8.89]	0.395	–		
Haiti	4.67^*^	**[1.31–16.66]**	**0.018**	**–**			2.88^*^	**[0.15–7.19]**	**0.024**
Colombia	0.57	[0.05–6.25]	0.645	1.08	[0.34–3.45]	0.891	0.67	[0.11–3.99]	0.658
Argentina	0.29	[0.02–4.64]	0.380	1.42	[0.59–3.42]	0.420	0.13^**^	**[0.03–0.58]**	**0.008**
Other countries in South America	1.26	[0.20–7.85]	0.802	2.09	[0.68–6.01]	0.200	0.44	[0.13–1.49]	0.186
Others	3.86	[0.45–32.77]	0.214	1.90	[0.66–5.47]	0.232	0.79	[0.31–2.04]	0.625
**Time of residencia (ref = 2010 or later)**									
2009–2000	0.25^*^	**[0.06–0.99]**	**0.048**	0.63	[0.22–1.77]	0.375	2.89^*^	**[1.04–8.04]**	**0.042**
1999 or before	0.45	[0.05–4.22]	0.481	1.66	[0.43–4.60]	0.324	11.04^****^	**[3.65–33.40]**	**< 0.000**
GOF test			< 0.000			< 0.000			< 0.000

*CI* confidence Interval; ^*^p value < 0.05; ^**^p value < 0.01; ^***^p value < 0.001; ^****^p value < 0.0001

Diverse variables were associated with health status among the Chilean population, including all demographic factors (Table 8). After adjusting by demographics, the lack of educational attainment was associated with a higher risk of NSPH, CM and DIS. In addition, being unemployed was associated with having NSPH (OR: 2.23; 95% CI: 1.72–2.89) and DIS (OR: 3.04; 95% CI: 2.49–3.70). The public health system affiliation was associated with higher odds of CM (OR: 1.83; 95% CI: 1.60–2.10) and DIS (OR: 1.24; 95% CI: 1.05–1.47). Meanwhile, those married/cohabitating were 58% less likely to have AL (OR: 0.42; 95% CI: 0.39–0.45), and 45% of having DIS (OR: 0.55; 95% CI: 0.52–0.58), while also presenting reduced odds of NSPH and CM. The highest level of income quintile was associated with 48% less chance of having NSPH (OR: 0.52; 95% CI: 0.46–0.59), as well as reduced odds of DIS (OR: 0.76; 95% CI: 0.69–0.85) and AL (OR: 0.73; 95% CI: 0.63–0.84). Among psychosocial factors, having social support was associated with 38% less odds of NSPH (OR: 0.62; 95% CI: 0.50–0.76), whereas social capital increased the odds of having CM (OR: 1.21; 95% CI: 1.15–1.27).

**Table 8:** Logistic regression models of health status outcomes by SDH in the Chilean born population

	Self-perceived bad health			Chronic morbidity			Disability			Activity limitations		
	OR	95% CI	*P* value	OR	95% CI	*P* value	OR	95% CI	*P* value	OR	95% CI	*P* value
**Demographic**												
Age	1.03^****^	**[1.03 - 1.04]**	**< 0.000**	1.06^****^	**[1.05 - 1.06]**	**< 0.000**	1.04^****^	**[1.04–1.04]**	**< 0.000**	1.06^****^	**[1.05–1.06]**	**< 0.000**
Sex (ref = male)	1.18^****^	**[1.13–1.24]**	**< 0.000**	1.54^****^	**[1.48 - 1.59]**	**< 0.000**	1.05^**^	**[1.02–1.10]**	**0.002**	1.18^****^	**[1.12–1.24]**	**< 0.000**
Ethnicity: (ref = no ethnicity)	1.16^**^	**[1.05 - 1.28]**	**0.003**	0.99	[0.93 - 1.05]	0.696	1.22^****^	**[1.13–1.31]**	**< 0.000**	1.17^***^	**[1.07–1.28]**	**0.001**
Marital status (ref = single)												
Married/Cohabitant	0.84^****^	**[0.78 - 0.89]**	**< 0.000**	0.94^**^	**[0.91 - 0.98]**	**0.007**	0.55^****^	**[0.52–0.58]**	**< 0.000**	0.42^****^	**[0.39–0.45]**	**< 0.000**
Separated/divorced/annuled	0.93	[0.84 - 1.03]	0.161	0.98	[0.93 - 1.04]	0.526	0.64^****^	**[0.60–0.69]**	**< 0.000**	0.54^****^	**[0.48–0.60]**	**< 0.000**
Widow	0.85^***^	**[0.77–0.94]**	**0.001**	1.06	[0.99 - 1.13]	0.107	0.90^**^	**[0.84–0.98]**	**0.009**	0.89^**^	**[0.81–0.98]**	**0.014**
Zone (ref = urban)	1.08^**^	**[1.00–1.17]**	**0.004**	1.04	[0.98–1.09]	0.172	1.00	[0.93–1.08]	0.971	1.04	[0.96–1.12]	0.384
GOF test			< 0.000			< 0.000			< 0.000			< 0.000
**Socioeconomic**												
Educational level (ref = none)												
University	0.34^****^	**[0.29–0.39]**	**< 0.000**	0.55^****^	**[0.49 - 0.62]**	**< 0.000**	0.24^****^	**[0.21–0.27]**	**< 0.000**	0.22^****^	**[0.18–0.27]**	**< 0.000**
Technical	0.38^****^	**[0.33–0.45]**	**< 0.000**	0.60^****^	**[0.53 - 0.69]**	**< 0.000**	0.27^****^	**[0.23–0.31]**	**< 0.000**	0.22	[0.18–0.27]	0.057
High School	0.46^****^	**[0.41–0.52]**	**< 0.000**	0.60^****^	**[0.54–0.68]**	**< 0.000**	0.29^****^	**[0.26–0.32]**	**< 0.000**	0.29	[0.26–0.34]	0.235
Primary	0.61^****^	**[0.54–0.69]**	**< 0.000**	0.74^****^	**[0.66–0.83]**	**< 0.000**	0.39^****^	**[0.36–0.44]**	**< 0.000**	0.38	[0.33–0.43]	0.604
Income quintile (ref = I lower income level)												
II	0.89^**^	**[0.82–0.97]**	**0.005**	1.00	[0.95 - 1.05]	0.913	1.00	[0.91–1.03]	0.310	0.93	[0.86–1.01]	0.083
III	0.84^****^	**[0.78–0.92]**	**< 0.000**	0.99	[0.94 - 1.05]	0.742	0.94	[0.88–1.00]	0.074	0.85^****^	**[0.77–0.93]**	**< 0.000**
IV	0.73^****^	**[0.66–0.81]**	**< 0.000**	0.97	[0.91–1.03]	0.270	0.83^****^	**[0.78–0.89]**	**< 0.000**	0.79^****^	**[0.71–0.88]**	**< 0.000**
V	0.52^****^	**[0.46–0.59]**	**< 0.000**	0.97	[0.90–1.05]	0.498	0.76^****^	**[0.69–0.85]**	**< 0.000**	0.73^****^	**[0.63–0.84]**	**< 0.000**
Occupation (ref = does not study)												
Unemployed	2.23^****^	**[1.72–2.89]**	**< 0.000**	0.80^****^	**[0.67 - 0.97]**	**< 0.000**	3.04^****^	**[2.49–3.70]**	**< 0.000**	0.04^****^	**[0.02–0.07]**	**< 0.000**
Studying	1.13	[0.87–1.46]	0.350	0.87	[0.72–1.05]	0.151	2.15^****^	**[1.74–2.67]**	**< 0.000**	0.19^****^	**[0.01–0.04]**	**< 0.000**
Employed	1.21	[0.93–1.57]	0.152	0.51^****^	**[0.43–0.62]**	**< 0.000**	1.44^****^	**[1.18–1.74]**	**< 0.000**	0.01^****^	**[0.01–0.02]**	**< 0.000**
Studying and work	1.61^*^	**[1.10–2.37]**	**0.015**	0.63^****^	**[0.50 - 0.79]**	**< 0.000**	2.11^****^	**[1.56–2.85]**	**< 0.000**	0.01^****^	**[0.00–0.02]**	**< 0.000**
GOF test			0.011			< 0.000			< 0.000			< 0.000
**Access to healthcare (ref = none)**												
Public health system affiliation	1.01	[0.82–1.24]	0.944	1.83^****^	**[1.60–2.10]**	**< 0.000**	1.24^**^	**[1.05–1.47]**	**0.010**	1.33	[0.99–1.79]	0.058
Private health system affiliation	0.85	[0.66–1.09]	0.195	1.91^****^	**[1.62–2.24]**	**< 0.000**	1.03	[0.85–1.24]	0.757	0.95	[0.68–1.31]	0.740
Other	0.91	[0.70–1.19]	0.470	1.65^****^	**[1.40 - 1.95]**	**< 0.000**	1.02	[0.83–1.25]	0.876	1.21	[0.85–1.74]	0.292
Doesn’t know	0.89	[0.67–1.19]	0.437	1.21	[1.00 - 1.47]	0.045	1.13	[0.90–1.42]	0.302	1.15	[0.80–1.68]	0.450
GOF test			0.002			< 0.000			< 0.000			< 0.000
**Psychosocial**												
Social support (ref = no)	0.62^****^	**[0.50–0.76]**	**< 0.000**	1.16	[0.98 - 1.37]	0.079	0.87	[0.72–1.05]	0.137	1.06	[0.83–1.35]	0.648
Social capital (ref = no)	0.80^****^	**[0.74–0.87]**	**< 0.000**	1.21^****^	**[1.15–1.27]**	**< 0.000**	0.97	[0.90–1.03]	0.319	0.83^****^	**[0.76–0.90]**	**< 0.000**
GOF test			0.384			< 0.000			0.001			< 0.000

*CI* confidence Interval; ^*^p value < 0.05; ^**^p value < 0.01; ^***^p value < 0.001; ^****^p value < 0.0001

### Findings on the healthy migrant effect

The odds of reporting each health outcome under study when being an international migrant (compared to Chileans as the reference) were calculated and progressively adjusted by each set of SDH (Table 9.). The unadjusted crude analysis revealed a healthy migrant effect, since being an immigrant was significantly associated with lower odds of presenting all health outcomes. After controlling for demographics, being an international migrant was no longer protective for NSPH and AL. However, after adjusting by socioeconomic covariates, only the association with CM remained significant. The subsequent models showed the presence of a healthy migrant effect for CM after controlling for access to health care and psychosocial factors. Being an international migrant was associated with 39% lower odds of chronic morbidity compared to Chilean population (OR: 0.61; 95% CI: 0.44–0.84; *P* = < 0.000).

**Table 9:** Logistic regression models of health outcomes if being an international immigrant sequentially adjusted by SDH

Health outcome	Model 1 Crude OR of being migrant			Model 2 Adjusted OR by demographics			Model 3 Adjusted OR by demographics + SES			Model 4 Adjusted OR by demographics + SES +access to health care			Model 5 Adjusted OR by demographics + SES +access to health care +psychosocial		
	OR	[CI95%]	***p***	OR	[CI95%]	***p***	OR	[CI95%]	***p***	OR	[CI95%]	***p***	OR	[CI95%]	***p***
Negative Self-perceived health	0.66^*^	**[0.45–0. 96]**	**0.031**	0.90	[0.62–1.33]	0.638	1.10	[0.71–1.60]	0.752	1.1	[0.71–1.64]	0.716	1.36	[0.81–2.28]	0.252
Chronic morbidity	0.30^****^	**[0.26–0. 35]**	**< 0.000**	0.43^****^	**[0.36–0.51]**	**< 0.000**	0.50^****^	**[0.42–0.61]**	**< 0.000**	0.54^****^	**[0.45–0.67]**	**< 0.000**	0.61^**^	**[0.44–0.84]**	**0.003**
Disability	0.45^****^	**[0.34–0. 60]**	**< 0.000**	0.66^**^	**[0.50–0.87]**	**0.004**	0.80	[0.57–1.05]	0.103	0.80	[0.58–1.08]	0.138	0.94	[0.55–1.62]	0.835
Activity limitations	0.44^**^	**[0.25–0. 80]**	**0.007**	0.80	[0.46–1.53]	0.560	1.10	[0.49–2.50]	0.812	1.2	[0.51–2.70]	0.719	2.17	[0.82–5.76]	0.121

*CI* confidence Interval; ^*^p value < 0.05; ^**^p value < 0.01; ^***^p value < 0.001; ^****^p value < 0.0001

## Discussion

Based on a secondary analysis of a nationally representative and anonymous survey conducted in Chile in 2017, we estimated the prevalence of a number of health outcomes among international migrants and the local population, as well as their association with demographic, socioeconomic, healthcare, psychosocial, and migratory SDH. After this, we tested the existence of the HME in each of these health outcomes. Results showed that migrants reported lower unadjusted prevalence of all the health outcomes under study compared to locals. In both groups, unemployment, affiliation to the health system and psychosocial factors were significantly associated with these outcomes. Among migrants, having lived in Chile for 20 years or more was associated with higher odds of reporting disability. Crude unadjusted models showed an apparent migrant’s health advantage regarding NSPH, CM, DIS and AL. However, after adjusting by demographics, socioeconomics, health care affiliation and psychosocial factors, being an international migrant only conferred protection for chronic morbidity. Previous evidence from the CASEN survey-2006 revealed a crude and adjusted by demographics advantage for any disability, health problem/accident and any chronic condition. In contrast to our findings, this advantage was no longer significant after controlling for socioeconomic and material covariates. Thus, the healthy migrant effect did not persist for any health outcome, highlighting the influence of poor socioeconomic status on health decline [17]. Other crude comparisons between international migrants and local population in South America, have suggested a probable existence of healthy migrant effect on chronic conditions. In Colombia, migrants from Venezuela had a lower self-reported prevalence of chronic diseases such as hypertension, cardiovascular diseases, diabetes mellitus and cancer than local population [34], similar to the smaller percentage of chronic conditions reported by Venezuelans in Peru [35]. Data from other sources such as hospital discharges, have revealed crude lower rates of CM in migrants residing in Chile [18]. Moreover, adjusted analysis on cancer hospital discharges also showed a potential advantage on this indicator [36].

The migrant advantage on CM could be explained by a positive selection, where those who decide to migrate are healthier than those who decided to stay. This better baseline health could be derived from access to a healthy diet and lower environmental risks, among other exposures in the country of origin, as well as their attitude towards long-term health by adopting healthier behaviors that might reduce risks factors for chronic diseases, while those who have medical conditions are more prone to returning [37]. This explanation might be complementary to the “cultural buffering” of the migrant’s group, whose norms reduce risky behaviors and promotes healthy decision making [38]. CASEN survey does not provide information related to behavioral factors; however, data from the Chilean national health survey (ENS 2016–2017) revealed elevated levels of alcohol consumption, smoking, sedentary lifestyle and low fruit and vegetable consumption among the general population. It also reported growing prevalence of chronic conditions such as type II diabetes mellitus, hypertension, dyslipidemia and obesity in the country [39]. Chile has experienced an epidemiological transition in the past century, moving away from infectious diseases and towards chronic conditions. In this process, the overall non-communicable disease (NCDs) burden has increased significantly, on average the Chilean adult population has four or more diseases [40] and NCDs have become the leading causes of death in Chile [41]. Compared to other countries in the Latin American region, Chile has a relatively higher rate of deaths caused by chronic diseases, which in turn contrasts with the lower self-reported rates of such conditions in the countries of origin of international migrants [42]. Therefore, the advanced epidemiological transition in Chile could yield a health gap between migrants and locals, that needs to be analyzed throughout the migrant life trajectory and with a SDH perspective.

The existing literature has suggested that HME disappears over time, meaning that the longer the length of stay in the receiving country, the higher the chance that migrants´ health assimilates to that of the native population [43]. This deterioration might be the result of cumulative exposures to health risk factors and other determinants, such as unhealthy behaviors that could be observed in the host society (e.g. smoking, alcohol consumption, poor diet), acculturative stress, discrimination and precarious living conditions [43, 44]. Our findings show a higher prevalence of unadjusted CM for migrants who have been living in Chile for over 20 years. However, time of residence was not associated with CM in the partially adjusted model. Thus, the exposure to diverse factors during the migration process does not seem to dissolve the advantage for chronic diseases seen in international migrants residing in Chile. In contrast, this protection might not apply for some long-term conditions such as disability, which was associated with a time of residence of 20 years or more. Findings suggest that even when migrants experience advantages in other health outcomes, they continue to face higher prevalence of disability compared to locals. There is also data suggesting that cumulative disadvantage resulting from social vulnerability could lead to occupational risks like high physical job demands, abuse and unsafe conditions that might play a role in the development of functional impairment [45]. There are some studies indicating that older migrants with a longer length of stay tend to display higher disability rates than both recent migrants and the local population [45, 46]. Furthermore, length of stay in the host country has also been inversely associated with self-perceived health which is in accordance with evidence reporting poor health perception in recent migrants [47]. However, studies on self-perceived health trajectories have shown that it could either remain stable or decline over time at a similar rate as locals. This evidence contrast to the inverse relationship commonly reported in cross-sectional studies [48].

Regarding the psychosocial resources, previous evidence have highlighted their protective role for migrants’ health [6, 8]. Our results showed both risk and protective associations between psychosocial factors and the health outcomes under study. Particularly, these factors were associated with increased odds of DI and CM but were protective for the remaining health outcomes. This dual effect has been previously suggested for migrant’s networks [49]. Depending on the composition of the networks, international migrants might be differentially exposed to healthy or risky behaviors (e.g., alcohol consumption determined by social situations, religious norms and ethnic identity) [50]. Meanwhile, social support might differ according to the migrant’s characteristics, migration-related factors, social contexts and types of supportive social ties. There is literature describing the “isolation paradox”, according to which migrants with poor social support were healthier than natives with similar isolation levels. The expected gradient between social support and good health has not always been seen in migrant population, as those with greater social support could also display poor health outcomes [51]. Moreover, the CASEN survey asks if the participant was under treatment in the past 12 months for CM. Thus, the association might result from the positive influence of social networks on health care utilization and health seeking behavior. Similarly, having health insurance could lead to increased access to diagnosis and treatment [52], which could explain the association between CM and healthcare affiliation, as these priority conditions are covered by the “Explicit Health Guarantees” of the Chilean health care system.

The present study contributes to the current understanding of the healthy migrant effect by comparing international migrants and the local population from a population-based secondary analysis. Our findings provide an insight of the influence of access to healthcare and psychosocial factors on migrant’s health status, beyond the influence of socioeconomic factors already described in previous research in Chile. This new evidence sheds light on plausible underlying mechanisms that produce health disparities between these populations and brings attention to its importance for health planning. However, the study has important limitations including the cross-sectional dataset that does not allow us to identify causal associations or detect changes over time of residence in the country among migrants. Estimations were based on self-reported data without medical confirmation and we used treatment for the past 12 months as a proxy of having chronic morbidity as available in the survey, since access to treatment in Chile requires a proven medical diagnosis. In addition, the CASEN survey does not provide data of behavioral and occupational risk factors to better understand the prevalence of long-term conditions. Moreover, it was not possible to include other psychosocial variables in the analysis, given that the survey did not ask for other indicators beyond social capital and social support, which restricted a more comprehensive analysis of this SDH. Similarly, we lacked additional migratory variables, such as reasons for migrating or risks involved during transit. Furthermore, it is possible that some migrants did not report that they were born abroad or those with irregular administrative status might have chosen not to participate. Therefore, migrants who experience greater social vulnerability might not be fully represented in this survey and their social determinants of health were not fully studied in this analysis. Future research should analyze migration trajectories, examining risks factors and health outcomes over time with longitudinal studies. The HME needs to be comprehensively tested by specific causes of morbidity from the SDH approach. Given the heterogeneity of the migrant population and the diversity of exposures they might face during the migration process, longitudinal studies in this matter could more effectively inform about the existence and relevance of the HME.

Our findings have practical implication towards inclusive public health responses. There is a number of SDH that could be targeted from a public health perspective, such as unemployment, type of affiliation to the healthcare system and psychosocial factors. These could be potentially modified by migrant-sensitive intersectoral actions and contribute to leaving no one behind in terms of healthcare and health status. Any initiative towards the social integration and the protection of the health of migrant communities should be based on equity and human rights approaches at both local and national level. For instance, it is necessary to foster social protection strategies and counteract socioeconomic vulnerability and poor living conditions that might result from unemployment and social marginalization regardless of immigration status. Public health efforts should also address barriers to healthcare affiliation and promote effective access and use of healthcare for everyone regardless of country of origin. Finally, the above-mentioned should be integrated with psychosocial support-based activities, while encompassing community based-interventions, intercultural competence in health care and evidence-based migration policies. These practical implications could be useful tools for encouraging collaborative alliances and policy making at regional level in Latin American region.

## Conclusions

The present study revealed an unadjusted advantage on health status among international migrants residing in Chile compared to the local population. Conversely, when a SDH approach was applied for adjustment, the healthy migrant effect disappeared for almost all health outcomes in our study. Being an international migrant remained protective for chronic morbidities, which might reflect the health gap from the advanced epidemiological transition experienced in Chile where NCDs represent the main public health issues. These findings bring attention to the need of further research on health disparities between international migrants and locals, while considering the diverse exposures during the migration process that could dissolve this health advantage over time. Our findings highlight the need to deepen the HME by cause-specific morbidity, particularly chronic conditions and their risks factors. This knowledge could be useful for health care practitioners and policy makers to develop a more comprehensive understanding of how variables like unemployment, affiliation to the health system and psychosocial factors may shape migrants’ health over time. It could be relevant for both policy and practice at health system level in Chile and more broadly in the Latin American region, especially for the purpose of “leaving no one behind in health protection”.

## Data Availability

The dataset analyzed during the current study is available in the Social Observatory website of the Ministry of Social Development http://observatorio.ministeriodesarrollosocial.gob.cl/encuesta-casen-2017. All data generated during this study are included in this published article.
